# How Do Culturally and Racially Marginalised (CaRM) Populations in Australia Cope with the Mental Health Impacts from “New Racism”? A Qualitative Descriptive Study

**DOI:** 10.3390/nursrep16030099

**Published:** 2026-03-16

**Authors:** Eric Lim, Shireesha Potla, Jaya Dantas, Takeshi Hamamura, Sender Dovchin, Stephanie Dryden, Ana Tankosić

**Affiliations:** 1 School of Nursing, Murdoch University, Murdoch, WA 6150, Australia; shireesha.potla@murdoch.edu.au (S.P.); stephanie.dryden@murdoch.edu.au (S.D.); 2 School of Population Health, Curtin University, Bentley, WA 6102, Australia; jaya.dantas@curtin.edu.au (J.D.); takeshi.hamamura@curtin.edu.au (T.H.); 3 School of Education, Curtin University, Bentley, WA 6102, Australia; sender.dovchin@curtin.edu.au (S.D.); ana.tankosic@curtin.edu.au (A.T.)

**Keywords:** Australia, coping strategies, culturally and linguistically diverse, culturally and racially marginalised, emotional safe space, mental health, new racism, qualitative research, resilience

## Abstract

**Background:** Australia’s increasingly multicultural landscape has seen a rise in culturally and linguistically diverse populations, many of whom face subtle and systemic forms of discrimination known as “new racism”. **Objective:** Underpinned by a person-centred and holistic framework, which recognises individuals as experts in their own lived experiences and emphasises strength-based, culturally situated understandings of well-being, this paper reports on a study that explores how culturally and racially marginalised diverse people in Australia cope with the mental health impacts of new racism. **Design:** A qualitative descriptive approach was employed in this study. **Participants:** Thirty participants from ten culturally and linguistically diverse communities participated in eight focus groups, providing rich insights into their lived experiences. **Methods:** Data were collected through semi-structured focus-group interviews conducted between March and June 2025. Data were analysed using Braun and Clarke’ method of thematic analysis. **Results:** Thematic analysis revealed four key coping strategies: (1) acceptance of immutable identity traits to foster resilience, (2) emotional ventilation within culturally safe spaces, (3) self-growth and empowerment through reflection and adaptive practices, and (4) assertive responses to racism when necessary. While some participants reported psychological distress, many demonstrated resilience and resourcefulness, challenging deficit-based assumptions often found in the existing literature. Findings underscore the importance of culturally responsive mental healthcare, including peer support, emotional safe spaces, and strength-based interventions. **Conclusions:** This study offers a holistic understanding of how culturally and racially marginalised people cope with new racism and its mental health impacts. The findings highlight the critical need for person-centred, culturally responsive, and equity-focused mental health support, providing actionable guidance for nursing practice and policy development.

## 1. Introduction

Australia has a significant migrant population, with 51.5% of Australians born overseas or having parents who were born overseas [1]. The results from the 2021 Census documented that 27.6% of Australia’s population was born overseas, and that 22.8% of people in Australia used a language other than English at home [2]. More specifically, the majority of migrants came from Asian countries, including China, India, Philippines, Vietnam, Malaysia, and Sri Lanka, contributing to more than 300 languages being spoken in Australian homes [2,3].

To bridge the shortfalls of skills arising from an aging population and low birth rates, Australia has been aggressively pursuing migrants, under its highly selective points skilled migration programs and annual migration skills demand list, to contribute to the national prosperity [4]. Many highly skilled and educated immigrants were granted entry to Australia through the skilled migration programs [4]. Between January 2015 and December 2024, the Australian Government reported that there were more than 1.5 million skilled migrants who had settled across all states and territories [2]. In addition, Australia also has a student migration pathway to encourage international students to pursue their university education [4]. This initiative has seen many international students gaining employment after they graduate and eventually settling down permanently in Australia [5].

Through the Refugee and Humanitarian Program, Australia has also offered refuge to 180,073 resettled refugees and asylum seekers in the 10 years to December 2022 [6]. Many of them were from countries that are experiencing civil conflicts, such as Iraq, Syria, Afghanistan, Myanmar, Iran, The Democratic Republic of Congo, and Pakistan. Refugees have experienced well-founded fear of being persecuted in their country of origin on the basis of their race, religion, nationality, political opinion, or membership of a particular social group [7]. Subsequently, Australia now has the highest share of foreign-born population as a share of the total population than in any other OECD country, except for Luxembourg and Switzerland [8].

## 2. Background

The rapid growth in migration has led to changes in demographic patterns in the Australian populations [9]. The term “culturally and linguistically diverse (CaLD)” is officially used in Australia to describe people with a cultural heritage differing from that of Anglo-Australians; who may speak English as an additional language; or who have other ethnocultural characteristics, such as country of origin, religious affiliations, and birthplace of parents [10]. We clarify that, due to a lack of a better term, “CaLD” will be used in this study and referred only to the individuals who are born overseas or born in Australia but have parents born overseas, international students, and refugee populations in Australia. However, we will use the term “culturally and racially marginalised (CaRM)”, as proposed by the Diversity Council of Australia (2023), when discussing new racism to refer to people who face marginalisation and discrimination due to their race and culture or background [11].

CaLD individuals who migrated to Australia are often faced with acculturation and acculturative stress arising from the socio-cultural differences from their country of origin [12]. This is particularly so if they are met with language barriers, linguistic discrimination, lower health literacy, unfamiliarity and difficulties navigating the institutional and financial resources, and/or a loss of social status [12]. Yet, it is important to note that CaLD migrants are influenced by their unique life events and backgrounds and face diverse challenges drawn from their lived experiences [12]. For example, the acculturation and acculturative stress experienced by skilled migrants and international students is expected to be significantly lower in severity when compared to refugees who have experienced significant trauma and flight [12]. This is because refugees are often met with a range of civil conflicts and violence, financial sacrifices, and stressful environments, either on route or upon arrival in Australia [12]. When resettling in Australia, they may experience difficulties in accessing healthcare that could result in poorer health and well-being, substandard housing that may lack basic sanitation, and limited financial means and prospects [13]. Moreover, in contrast to the 96 percent of skilled migrants in Australia who speak English well, or very well, this drops to 71 percent of humanitarian migrants who are permanent residents, falling further to 56 percent for humanitarian migrants who have lived in Australia for five years or less [14].

Nevertheless, regardless of an individual’s English ability, there is increasing evidence that CaLD people who migrate to Australia are faced with various types of racism. This is due to their non-standard racial and ethnic classifications, language practices, and ethnic accents, alongside non-Anglo Western cultural ways, being categorised as cultural and identity characteristics that differ from the mainstream society [4]. However, unlike the historic overt form of racism that is considered as socially undesirable and repulsive, CaLD people are now faced with more subtle, insidious, and covert forms of prejudice and discrimination [15]. “New racism” [16] is a term used in modern multi-cultural societies to refer to a culture-based discrimination of migrants who differ from the prescribed standard. New racism also recognises that different identity markers—culture, ethnicity, race, language, and/or religion—intersect in different ways, leading to ideological assumptions that migrants are not making efforts to solve their own problems, and a failure to acknowledge that migrants may have additional needs for government assistance [17]. Other forms of new racism include devaluing the overseas qualifications and experiences of CaLD skilled migrants from non-English speaking countries, assuming individuals have “poor” English language proficiency based on their first and/or last name and accent, placing greater pressure on people to prove their worth, employing people in roles that do not match their qualifications, and offering lower salaries compared to others in the same job [4].

People with foreign-sounding names were frequently found to have received fewer call backs for job interviews when applying for jobs [4] or were offered fewer positions that matched their pre-migration skills and experiences when compared to people with English-sounding names [16]. As such, the prevalence of new racism in Australia has led many CaRM people to resort to a practice called “CV whitening”, such as using “fake” English names or altering one’s name, to avoid anticipated labour discrimination by minimising or downplaying their ethno-racial clues in job applications [18]. In social situations, it is also common for CaRM people to feel that they are socially excluded. Importantly, even if a CaRM person speaks English well and has the required linguistic resources to navigate interactions, a major issue is the illegitimacy placed on the variety of English they use, which indicates the problem is with their cultural background, race, or ethnicity, rather than any actual communicative issues [19]. In summary, new racism is deeply rooted in the normative and often invisible nature of Anglo-Whiteness as the standard against which all other identities, cultures, and languages are measured [20]. It manifests not only as overt prejudice or discrimination but also as systemic and subtle biases that devalue, marginalise, and pathologise cultural and linguistic diversity. This form of racism is informed by deficit-based perceptions that frame non-dominant ways of being, speaking, and knowing as inferior, inadequate, or problematic [16]. By privileging dominant cultural norms, new racism operates both at individual, systemic, and institutional levels, shaping social, educational, and professional contexts in ways that constrain opportunities for those whose identities do not align with the standards of the dominant group—in the Australian context—Whiteness.

## 3. Purpose of This Study

Experiences of new racism can have profound and far-reaching effects on an individual’s mental health. Exposure to new racism has been shown to contribute to low self-confidence, diminished self-esteem, psychological trauma, emotional dysregulation, shame, humiliation, stress, insomnia, and even suicidal ideations [21], all of which can significantly contribute to an onset of a mental illness [15]. Despite the clear mental health implications, there remains limited understanding of how CaRM people actively cope with these experiences. Most existing research frames the CaRM population in terms of vulnerability or barriers, emphasising deficits rather than recognising the potential resources, resilience, and culturally relevant strategies that CaRM people may draw upon to navigate mental health challenges [22]. This gap in knowledge limits our ability to develop culturally responsive support mechanisms and interventions that effectively address the needs of CaRM populations [23].

Therefore, the purpose of this study was to explore the coping strategies employed by CaLD people in Australia in response to the mental health impacts of new racism. By focusing on their lived experiences, this study aims to show both the challenges and the adaptive resources that shape how CaLD people maintain their psychological well-being in response to new racism, providing insights that may inform culturally sensitive mental health practice and policy.

This study is underpinned by a person-centred and holistic framework [24], which recognises individuals as experts in their own lived experiences and emphasises strength-based, culturally situated understandings of well-being. This study’s approach is aligned with the United Nation (UN)’s Sustainable Development Goals (SDG) 3—ensure healthy lives and promote well-being for all at all ages; and SDG 10—reduce inequality within and among countries [25]. This study’s approach is also aligned with the UN International Organization for Migration’s Global Compact for Migration Objectives 16—inclusion and social cohesion; 17—eliminate discrimination; 18—skills development and recognition; and 22—social protection [26].

## 4. Materials and Methods

A qualitative descriptive research approach was used in this study to explore the impacts of new racism on CaRM people’s mental health and their coping strategies. This approach was particularly well-suited to our research, as we sought to capture participants’ complex experiences through in-depth and detailed insights. By adopting a qualitative descriptive research approach, we were able to remain close to the data, minimising the imposition of pre-existing theoretical frameworks or extensive interpretive analysis, thereby preserving the authenticity of participants’ voices. This approach also allowed for a clear, straightforward description of phenomena as experienced by participants, while facilitating the identification and organisation of patterns or recurring elements in the data. The process involved cataloguing information into coherent themes, which then provided meaningful insights into the ways in new racism affects mental health and how CaRM people develop resilience and coping mechanism in response to these [27].

This study was part of a Healthway-funded project that received ethics approval from Curtin University Human Research Ethics Committee (approval number HRE2024-0576) and Mudoch University Human Research Ethics Committee (Project No. 2025/084).

Ethical principles were upheld throughout all stages of this study. All participants received written and verbal information about the purpose, procedures, potential risks, and voluntary nature of the research before providing informed consent. Confidentiality was ensured by de-identifying transcripts and securely storing all data in accordance with institutional requirements. Given the sensitivity of discussing experiences of racism, the research team took care to create culturally safe and supportive environments during focus groups, and participants were reminded that they could pause, skip questions, or withdraw at any time without any consequence. The research team also adopted culturally responsive practices by respecting linguistic diversity, acknowledging power imbalances, and minimising any potential for re-traumatisation.

### 4.1. Participants and Sampling

Purposeful sampling method was used in this study to recruit approximately 3–5 members for each focus group from 10 CaLD communities. The CaLD communities included Bhutanese, Chinese, Indian, Japanese, Filippino, Kenyan, Malawian, Mongolian, Singaporean/Malaysian, and Vietnamese. Participants were recruited through the Consumer Advisory Group, professional networks, universities’ student guild, and the local community centres.

Participants were provided with a participant information sheet with a consent form to inform them of the purpose and nature of this study. Interpreter services were provided to CaLD participants who needed clarification in their preferred language. The inclusion criteria included (i) being 18 and above, (ii) being a CaLD migrant or having a parent who is a first-generation migrant, (iii) have experienced cultural and/or racial discrimination, and (iv) being willing to provide informed consent to participate in the focus group voluntarily. Individuals who expressed an interest in participating in this study were invited to join the focus group with other participants of a similar CaLD background for a discussion.

### 4.2. Data Collection

Data were collected through semi-structured focus-group interviews conducted between March and June 2025. Demographic information of the participants was obtained from the participants prior to the discussion that was audio recorded. During data collection, participants were encouraged to use English and their heritage languages to articulate their experiences in a meaningful and accurate way. A researcher or member of the Consumer Advisory Group that spoke the same language of the participants attended the focus group to assist in comprehension and to ensure the natural flow of the conversation. At the end of data collection, transcripts that contained parts in a language other than English were translated to English by a research member who spoke that language. Data collection in this study was guided by data saturation and ceased when subsequent focus-group discussions provided no new information [28].

### 4.3. Data Analysis

Focus-group data were analysed using Braun and Clarke’s method of thematic analysis to construct an understanding of the phenomenon being explored [29]. The process of coding and construction of themes were performed by the 1st and 2nd authors, who are experienced in qualitative research, and were shared with all the authors to enhance and ensure the credibility of findings. Trustworthiness of the data analysis process was ensured through investigator triangulation between the 1st and 2nd authors, and with peer review by the research team (six come from CaLD migrant background and one comes from Anglo-Australian background) to detect any bias or inappropriate subjectivity in the authors’ interpretations of the data.

During the analysis stage, all recorded focus-group data were transcribed verbatim and repeatedly read to identify emerging themes relevant to the objectives. Secondly, the identified sentences and segments were compared, and those with associated characteristics were grouped to build potential themes [29]. Finally, the constructed themes were compared to allow meanings to emerge, and this process enabled the authors to write up the findings. To achieve dependability, this study was reported in accordance with the COREQ Qualitative Checklist for Interviews and Focus Groups [30]. Descriptive statistics were used to analyse and present the collected demographic data.

## 5. Results

A total of eight focus groups were conducted with thirty participants. Three of the focus groups were conducted face-to-face, and the other five online via Microsoft Teams. The shortest focus group lasted for approximately 40 min, and the longest focus group lasted for approximately 85 min. The mean time of all the focus groups was 63 min.

Seven participants identified themselves as males, and 23 participants identified themselves as female. Five of the participants (16.7%) were aged between 18 and 21, seven of them (26.7%) were aged between 20 and 29, six of them (20%) were aged between 30 and 39, six of them (20%) were aged between 40 and 49, and six of them (20%) were aged 50 and above. Additionally, 15 (50%) of the participants were citizens, 12 (40%) of them were international students, and 3 (10%) of them were permanent residents. Twenty-three of the participants (76.7%) used English (including those who used both their first language and English) at home, and 18 of the participants (60%) have lived in Australia for more than 10 years. The demographic information of the participants is presented in Table 1.

Table 2 illustrates the participants’ personal experiences with speaking English, experience with new racism, and its mental health impacts. The majority of the participants felt confident speaking in English (93.3%) but have been treated differently because of the way they used English (73.3%) or their accent when speaking English (83.3%). Slightly more than half of the participants felt that they had been treated differently because of their skin colour (63.35%); had experienced situations where others lacked understanding of their cultures (60.0%); and developed negative feelings when speaking in English (56.7%).

Four themes emerged during data analysis and revealed the participants’ perspectives of the strategies that CaLD people in Australia utilised to cope with the mental health impacts of new racism.

### 5.1. Theme 1—Acceptance of Immutable Identity Traits to Foster Resilience

Theme 1—“acceptance of immutable identity traits to foster resilience”—reflects participants’ acknowledgement that aspects of their identity, such as culture, language, race, or ethnicity, are inherent and beyond their control. Participants described accepting these immutable characteristics as a strategy to manage the psychological impacts of new racism, recognising that self-acceptance could buffer against internalising racist attitudes or self-blame. By framing their identity as something innate and unchangeable, participants were able to cultivate a sense of resilience and self-preservation, focusing on adapting to external challenges rather than altering themselves to fit dominant societal expectations. This perspective also highlights the tension between societal prejudice and personal agency, presenting how individuals navigate a world where their inherent difference can become targets of bias, while simultaneously asserting their right to exist authentically without self-modification.

As one of the participants who was discriminated against at work stated, “*I know I’m saying it wrong according to Australian people… [sic] at some level, people are going to judge you, at the end of the day, they’re going to judge you*” (P11, Indian, female, aged 20 to 29). Therefore, most of the participants said that they would try not to internalise the stigma so that it would not have a significant impact on their mental health:

“*I really don’t think much about it because it’s [racism] not something I can control like it’s something that I’ve been born with. So, there’s nothing I can do about it. It’s more of like if I face it, I just need to go through it, through day like, I don’t think much about it in a way*”.

(P1, Bhutan, female, aged 20–29)

Another participant who was discriminated against for the English variety she speaks shared the following:

“*Indian accent is different. And I knew… I’m going to face racism. My accent is going to be different. I can’t speak English that confidently… So, yeah, it made me feel insecure at some point. It made me hesitate to ask questions in public and talk in public. But, you know, I don’t care. I’m like, yeah, it’s our accent. I can’t do anything It’s our accent; it’s the way we speak. We can’t change it. We can adapt it, but we can’t change it*”.

(P11, Indian, female, aged 20 to 29)

The participants who contributed to this theme elaborated that they are more resilient than what the public perceived them to be. “*We are very strong. We have built the resilience for so long, and coming from Africa, for example myself… met with difficult things, you know issues*” (P14, Malawian, male, aged 40 to 49); “*I already accepted everything, accepted my little English, I can’t hide it*” (P19, Mongolian, female, aged 40 to 49). As such, the participants did not perceive the experience of new racism to have a significant impact on their mental health. “*I wouldn’t say it as mental illness or anything to do with a psychological distress, it’s just mainly, just maybe, I would say, a stressful situation*” (P23, Kenyan, female, aged 50 and above); “*I may say these are stressful, but they don’t go to the extent where I would need to seek attention, uh seek medical support*” (P14, Malawian, male, aged 40 to 49); “*I can get distress maybe for a few days and then it goes away. I think from how I’ve grown up… my resilience is ok*” (P15, Malawian, female, aged 40 to 49).

Nonetheless, some of the participants felt that being uniquely different could still be emotionally and psychologically taxing. One of the participants lamented that, “*I’ve to just go through that phase, and… someday they’re going to understand us*” (P13, Indian, female, aged 18 to 19). Another participant who expressed that she is learning to accept her unique differences stated the following:

“*Accepting is really challenging… It’s difficult because people are treating you like this… I never think about that before coming here, so still in the process of accepting this… to accept all the challenge to move forward*”.

(P20, Mongolian, female, aged 30 to 39)

Additionally, some of the participants felt that being uniquely different means that they would need to learn to be “*more cautious*” (P29, Singaporean, female, aged 30 to 39) to avoid being discriminated against for using English. For example, a participant who had lived in Australia for more than 36 years and spoke English fluently stated the following:

“*It is like the extra burden of having to think before you do… you know, everything you do, you just have to be careful of what you say before you say it. Like, oh, is that OK? Will it come off the wrong way?*”.

(P16, Malaysian, female, aged 30 to 39)

### 5.2. Theme 2—Emotional Ventilation Within Culturally Safe Spaces

The second theme is “emotional ventilation within culturally safe spaces” and highlighted the perspectives of the participants that they may ventilate about their experience of new racism to de-escalate its mental health impacts. This theme was likely contributed by participants who experienced support from their CaLD communities living in Australia. Based upon the participants’ conversations, there was a sense of comfort, comradeship, and shared understanding that they could talk to other CaRM people about their negative lived experiences. “*It doesn’t even matter whether you know the person’s name or not, we just talk about it, so there are ways of being able to ventilate and share our experiences*” (P23, Kenyan, female, aged 50 and above); [sic] “*it helps, because my friends always give back the same energy and they always tell what I want to hear*” (P2, Bhutanese, female, aged 20 to 29); “*When I first experienced it, my friends and I, we were together, so we just talked to each other, but pretty much that was it… and it just makes it little bit better*” (P1 Bhutanese, female, aged 20 to 29). One of the participants shared that when a CaLD individual ventilates to another CaRM individual, “*you’re talking in a safe space and you will never, never until the day you die, you will never hear it outside that space of ventilating*” (P23, Kenyan, female, aged 50 and above).

However, there was a consensus among the majority of the participants that they were unlikely to ventilate to a family member: “*it’s such a difficult subject to talk about I find, with my aunts and uncles, my parents*” (P29, Singaporean, female, 30 to 39) [sic] “*I think I wouldn’t be talking with my parents about it. I don’t want them to worry*” (P1 Bhutanese, female, aged 20 to 29). Another participant who shared the same sentiments said that “*when you get home, you have to put a brave smile on to your parents, because they don’t know what’s going on with your life*” (P24, Filipino, female, aged 18 to 19). They would also be unlikely to ventilate to a friend living in their home country about their negative lived experiences as “*they didn’t experience this, and can’t fully understand I guess… and so, I just tell then the bright thing about like Australia and thing*” (P20, Mongolian, female, aged 30 to 39).

### 5.3. Theme 3—Self-Growth and Empowerment Through Reflection and Adaptive Practices

The third theme—“self-growth and empowerment through reflection and adaptive practices’ captures participants”—places emphasis on personal growth and self-empowerment as strategies to cope with the mental health impacts from new racism. Participants described deliberately cultivating resilience, learning from adverse experiences, and developing inner strength as ways to navigate the emotional challenges posed by new racism. This theme often involved reflecting on past encounters, reframing negative experiences as opportunities for growth, and strengthening their sense of self-worth and confidence. By focusing on self-development, participants were able to maintain a sense of agency in situations where systemic racism limited their external control. This theme highlights how coping with new racism extends beyond immediate responses, encompassing proactive and ongoing efforts to build psychological resilience, assert personal identity and transform experiences of racism into sources of empowerment and self-assurance. As P27 explains,

“*You have to learn to do things by yourself. I think going back to the negatives experiences, it’s a bad thing, but it’s important to reflect upon the negative experiences so you can use them to improve and make the positives more prevalent than the negatives… it’s kind of sad that we have to face the negatives in order to, you know, find the positives*”.

(P27, Filipino, male, aged 18 to 19)

The participants shared that when they focus on self-growth, they are more likely to think positively instead of dwelling in the negative lived experience:

“*There is a good side of Australia, you know, even though there is racism. The health care is free; bus and train are free for the moment… If I’m still in the Philippines, I know that I’m not going to experience any racism because I barely see anyone… [But] I would not have made friends, multiculturally, if I was not in Australia. So, I’m glad I’m able to experience the positive side. I’m going to keep looking at the positive sides only, because if I look at negative that’s when my mental health goes crazy*”.

(P24, Filipino, female, aged 18 to 19)

Some of the participants shared that they would engage in more self-care activities to support their mental wellness. One of the participants shared that she would do something to relax every time she experienced discrimination. “*I was walking a lot. Walking made me calm down and settle down. Some meditation helped me too*” (P22, Mongolian, female, aged 50 and above). Another participant who is more religious found that she would “*pray to God and go in church. More like religion things, read Bible, and. Yeah. I just tell everything to God. My God, that’s what I do*” (P20, Mongolian, female, aged 30 to 39).

Notably, the participants highlighted that the focus of self-growth is not only important for them to maintain their mental health “here and now” but can also empower them to become more resilient when faced with new racism in the future. As one of the participants claimed,

“*It didn’t really affect my mental health in the long term, but in the short term, of course. But we just make ourselves strong so that their opinions do not affect us… [sic] in the context of racism, by experiencing day by day, you become stronger*”.

(P13, Indian, female, aged 18 to 19)

Another participant who felt that she had experienced self-growth after her experience of new racism concluded, “*I think we are prepared now. Like, things have happened to us. Now we are prepared, now we are not the same. All of that, you become stronger, and you have no fear of anything now*” (P12, Indian, female, aged 20 to 29). This participant’s reflection suggests a sense of post-traumatic growth, portraying experiences of new racism as developing resilience and personal growth, such as “you become stronger” or “no fear of anything”. However, this framing poses the risk of individualising the impact of systemic racism, potentially obscuring the ongoing structural and emotional burdens it imposes. The reflection also reflects a coping mechanism that emphasises stoicism, possibly masking continued vulnerability. Intersectional factors such as the participant’s identity as a young Indian woman, further shape how resilience is experienced and expressed. While empowering, such accounts should be interpreted critically, recognizing both personal growth and the persistent inequities underlying these experiences.

### 5.4. Theme 4—Assertive Responses to Racism When Necessary

The final theme—“assertive responses to racism when necessary”—reveals participants’ recognition that, in certain situations, responding directly to racist behaviour is necessary to assert their dignity and protect their well-being. Participants describe this form of resistance as a measured response, typically employed only after other coping strategies, such as ignoring, avoiding, or seeking social support, have been exhausted. The decision to “*clap back*” was framed as a last resort, triggered when they perceived that their tolerance had been exceeded or when the discriminatory behaviour became too blatant or harmful or ignore. This theme points out the delicate balance participants navigate between self-preservation and active resistance, reflecting both the emotional labour involved in managing encounters with new racism and agency exercised in choosing when and how to respond. For example, one of the participants who was discriminated against for eating food that was unique to her culture said that “*sometimes I just clap back, like, oh your food doesn’t taste like anything [laughter]. Just like counter racism your racism*” (P25, Filipino, male, aged 20 to 29). For the participants, there was a consensus that the suppression of intense negative emotions could be detrimental for one’s mental health. [sic] “*It’s so tempting to not say anything, but if you just ignore it, it’s just going to get you’re your feels… You’re going to get to that anger, and you want to punch the guy… Oh my gosh, you know… Rather than like that, you speak up*” (P24, Filipino, female, aged 18 to 19).

In addition, some of the participants felt that all CaRM people should stand up for themselves when faced with new racism so that the perpetrator will learn to stop doing it. As one of the participants who felt that people are most likely ignorant if they are racist shared, “*I just assume that they didn’t know any better. So, if someone is racist, that means they’re not as educated*” (P3, Bhutanese, male, aged 20 to 29). This was backed by another participant who stated that

“*I think some people just think racism is about talking to a Black people to say you’re Black. But there is more, and I think if people are made aware that if you speak in this way to another person, the person will not feel well because you’re discriminating them*”.

(P14, Malawian, male, aged 40 to 49)

## 6. Discussion

This paper presented results and findings from a qualitative study that explored the coping strategies that CaRM people in Australia utilised to cope with the mental health impacts of experiencing new racism. We interpret the findings through a person-centred and holistic framework, which focused on the participants’ lived experience to understand how they cope with the mental health impacts from new racism. The person-centred and holistic lens prevented us from pathologising distress, and instead highlighting capability, choice, and context [24]. Therefore, the findings of our study are significant to bridge the current knowledge gap in the literature, as previous studies were found to be focused on the psychosocial deficits of CaRM people in Australia to cope with their negative lived experiences [31], or on the types and mental health impacts of new racism [31].

Unsurprisingly, much of the existing literature on CaRM people and the mental health impacts of new racism were published during the COVID pandemic [32]. As such, the intention of their research was placed on highlighting the negative lived experiences of CaRM people in Australia. Nevertheless, this could have significantly contributed to an increasingly negative image of this population [5]. In fact, research that solely adopts a deficit-focused, rather than a holistic, view of CaRM people and their psychosocial capabilities to cope with mental health issues [33] is inherently a form of discrimination, as it could potentially contribute to a vicious cycle that perpetuates stigma of CaRM people. One example of this is the belief that CaLD people have poorer mental health literacy when compared to the non-CaLD people in Australia. As such, the findings of our study are significant to provide an in-depth and holistic view of CaLD people in Australia.

The findings of our study revealed that CaRM people may use a range of coping strategies to navigate the mental health impacts of experiencing new racism. For example, it was found that they may learn to accept their unique difference from mainstream society to mitigate negative thoughts and emotions when they are discriminated against. Research evidence suggested that individuals who have the ability to cope with discrimination positively were more likely to utilise adaptive coping styles, and this was closely related to them having a more positive mental health outcome [32]. Nonetheless, Huynh and Lee found that individuals would still need to achieve a positive reinterpretation of their lived experiences to prevent ending up in emotional suppression [34]. Positive reinterpretation is the reframing of stressful situations to make it more tolerable to reduce the intensity of negative thoughts and emotions [34], and this brings us to the importance of having an emotional safe space for CaRM people.

The availability of an emotionally safe space, which refers to linguistically inclusive zones where CaRM people can feel safe to share lived experiences, emotional struggles, and personal narratives, was found to be an adaptive coping strategy by the participants in our study. Consistently, the existing literature also reported that having an emotional safe space to share or vent negative lived experiences with another individual who has faced, endured, and overcome the same has the effects of validating and reducing the person’s psychological distress, and was associated with positive reinterpretation, positive thinking, and self-growth [34].

It was found that there was a reluctance for CaRM people to share their negative lived experience with their family members and close friends. While most of the existing literature showed that family members and close friends play an important role of supporting CaRM people to overcome negative impacts on their mental health [35], it was found that they may avoid sharing their experience with new racism, not wanting to worry loved ones or because they felt their family and friends would not understand. This finding may resonate with prior studies, which suggested that such avoidance serves to prevent generalising their negative feelings and worrying them [9].

The findings of our study are like that of previous studies: CaRM people in Australia are likely to focus on self-growth to cope with their experience of new racism [5,36]. The focus on self-growth would require CaRM people to be hopeful that they would one day become a better version of themselves [35]. As such, CaRM people are likely to use adaptive coping strategies, such as engaging in self-help activities [5]. Subsequently, individuals who experienced self-growth are likely to find themselves becoming increasingly more confident, resilient, and resourceful when facing these challenges [5]. Consequently, self-growth was regarded as the most effective, practical, and least expensive method that individuals who experienced discrimination could use to cope with the mental health impacts and to prevent long-term problems [5].

Nevertheless, the findings of our study also revealed that CaRM people may also fight back or stand up for themselves as a last resort if they felt overwhelmed by their experience of new racism. The findings of our study resonated with that of previous research which showed that individuals could become aggressive if they feel they experienced overwhelming levels of stress [31]. As such, CaRM people may display aggression as a defence mechanism to instrumentally deflect or stop racial discrimination to reduce its negative impact on their mental health [37].

### 6.1. Relevance to Clinical Practice

The findings of our study are useful to increase nurses’ and other health professionals’ understanding of CaRM people in Australia and to promote more culturally responsive and appropriate care. Evidence generated from our study on the adaptive coping strategies that CaRM people perceived as critical for them to maintain their mental health can also support nurses and health professionals to identify more effective interventions.

Firstly, knowledge that CaRM people are likely to focus on self-growth, we propose that nurses and health professionals plan and implement more educational activities and strength-based activities to empower them with the resources to cope with the mental health impacts of experiencing new racism [36]. For example, the use of adaptive coping strategies such as recognising and responding to new racism; emotional literacy and validation; positive coping mechanisms; advocacy and help-seeking to support CaRM people to recognise new racism and its impacts on their mental health [5]; psychotherapy to support them to achieve positive reinterpretation of their negative lived experience; and recovery-focused care to allow the person to self-determine what they need to achieve their personal recovery [37].

Secondly, having an emotional safe space was an important element for CaRM people to talk about their lived experience and use positive reinterpretations of their negative thoughts and emotions. This was identified as a precursor for CaRM people to develop positive thinking and to focus on their self-growth. As highlighted by the participants, the construction of an emotional safe space would require having a person who shared their culture and have had similar lived experiences, while maintaining some distance from family and friends. Therefore, the findings of our study highlighted the importance of having CaRM peer support workers in healthcare settings in the pursuit of culturally responsive and appropriate care [38].

Finally, nurses and health professionals caring for CaRM people assessed as having risk for aggression should always endeavour to use culturally appropriate language and support CaRM people to experience a therapeutic milieu [9]. The use of culturally appropriate language when caring for CaRM people may also be critical to mitigate any interpersonal and situational conflicts that could consequently lead to unwarranted behaviours such as aggression in healthcare settings [37].

### 6.2. Limitations of This Study

Like all research studies that use a qualitative research approach, the aim of our study was to obtain an in-depth understanding of a phenomenon of interest and to provide examples of real stories of real people. As such, the findings may only be a representation of CaRM people in Australia who participated in our study. Additionally, the generalisability of the findings in our study was also limited by the intersectionality of CaRM people in Australia. Nevertheless, the findings of our study provided an increased understanding of the potential strategies that CaRM people in Australia may use to cope with the mental health impacts of experiencing new racism.

## 7. Conclusions

This study employed a person-centred and holistic framework to examine how CaRM people in Australia experience and respond to the mental health impacts of new racism. Our findings contributed foundational insights to the strengths and resilience of CaRM people to cope with challenges such as new racism and their impacts on their mental health.

Our findings underscore the need for healthcare services to incorporate emotional safe spaces where CaRM individuals can safely express and process experiences of racism, ensuring access to interpreters, and strengthening organisational capacity for culturally responsive and anti-racist practice. Our findings also underscore the need for health professionals to consider cultural identity, language, and lived experience in assessment and care planning.

Finally, future research should prioritise co-designed, community-embedded interventions with CaRM people to test the effectiveness of the coping strategies identified in this study. Longitudinal and mixed-methods designs would help examine how coping strategies evolve over time, particularly in relation to changing migration contexts, generational transitions, and systemic barriers such as linguistic discrimination. There is also a need for intervention studies that evaluate how organisational practices, such as providing an emotional safe space, having an interpreter involved throughout their care and treatment, and integrating CaLD peer workers, shape mental health outcomes.

## Figures and Tables

**Table 1:** Demographic information of the participants (N = 30).

Participant Number	Country of Origin	Gender	Age Group	First Language	Uses English at Home	Highest Attained Education Level	Years Living in Australia	Residency Status in Australia
1	Bhutan	Female	20 to 29	Dzongkha	Yes	Vocational	2	International student
2	Bhutan	Female	20 to 29	Bhutanese	Yes	Vocational	2	International student
3	Bhutan	Male	20 to 29	English	Yes	Vocational	1	International student
4	China	Female	50 and above	Mandarin	Yes	Postgraduate	25	Citizen
5	China	Male	40 to 49	Mandarin	Yes	Postgraduate	27	Citizen
6	China	Male	50 and above	Mandarin	No	Postgraduate	25	Citizen
7	China	Female	40 to 49	Mandarin	Yes	Postgraduate	16	Permanent resident
8	Japan	Female	50 and above	Japanese	Yes	Vocational	20	Permanent resident
9	Japan	Female	50 and above	Japanese	Yes	Undergraduate	10	Permanent resident
10	Japan	Female	30 to 39	Japanese	Yes	Vocational	5	International student
11	India	Female	20 to 29	Hindi	Yes	High school	4	International student
12	India	Female	20 to 29	Hindi	Yes	High school	1	International student
13	India	Female	18 to 19	Punjabi	No	Undergraduate	1	International student
14	Malawi	Male	40 to 49	Chichewa	Yes	Postgraduate	11	Citizen
15	Malawi	Female	40 to 49	Tumbuka	Yes	Postgraduate	10	Citizen
16	Malaysia	Female	30 to 39	English	Yes	Undergraduate	36	Citizen
17	Mongolia	Female	30 to 39	Mongolian	No	Postgraduate	1	International student
18	Mongolia	Female	30 to 39	Mongolian	No	Postgraduate	2	International student
19	Mongolia	Female	40 to 49	Mongolian	No	Postgraduate	2	International student
20	Mongolia	Female	30 to 39	Mongolian	No	Postgraduate	2	International student
21	Mongolia	Female	20 to 29	Mongolian	No	Postgraduate	2	International student
22	Mongolia	Female	50 and above	Mongolian	Yes	Postgraduate	23	Citizen
23	Kenya	Female	50 and above	Swahili	Yes	Postgraduate	20	Citizen
24	Philippines	Female	18 to 19	Tagalog	Yes	High school	10	Citizen
25	Philippines	Male	20 to 29	Tagalog	Yes	Vocational	14	Citizen
26	Philippines	Male	18 to 19	Tagalog	Yes	Vocational	11	Citizen
27	Philippines	Male	18 to 19	Tagalog	Yes	Undergraduate	16	Citizen
28	Philippines	Female	18 to 19	Tagalog	Yes	High school	10	Citizen
29	Singapore	Female	30 to 39	English	Yes	Undergraduate	36	Citizen
30	Singapore	Female	40 to 49	English	Yes	Postgraduate	18	Citizen

**Table 2:** Personal experiences with speaking English, experience with new racism, and its mental health impacts.

Questions	(%)
Do you feel confident speaking in English?	
Yes	93.3
No	6.7
Have you ever felt that you were treated differently because of your English?	
Yes	73.3
No	26.7
Have you ever felt that you were treated differently because of your accent when speaking in English?	
Yes	83.3
No	16.7
Have you ever felt that you were treated differently because of your skin colour?	
Yes	63.3
No	36.7
Have you ever felt that you were treated differently because you speak another language?	
Yes	46.7
No	53.3
Have you been in a situation where you felt hesitant, stressed, anxious, angry, sad, or frightened because you were speaking in English?	
Yes	56.7
No	43.3
Have you been in a situation where you felt stressed angry, sad, or frightened because you were speaking in your first language?	
Yes	53.3
No	46.7
Have you been in a situation where you felt stressed, angry, sad, or frightened because of others’ lack of understanding of your culture?	
Yes	60.0
No	40.0
Have you ever sought help to support or improve your mental health and well-being?	
Yes	40.0
No	60.0

## Data Availability

Data collected in this study are not publicly available due to privacy and ethical restrictions but can be made available upon reasonable request from the corresponding author.

## Public Involvement Statement

CaLD people in Australia were involved in this research through their participation in the focus groups.

## Guidelines and Standards Statement

This manuscript was prepared following the procedures outlined in Strengthening the Reporting of Observational Studies (STROBE). A complete list of reporting guidelines can be accessed via the equator network: https://www.equator-network.org/.

## Abbreviations

The following abbreviations are used in this manuscript:

CaLD	Culturally and linguistically diverse
CaRM	Culturally and racially marginalised
COVID	Coronavirus disease
